# A National Survey of Mentoring Practices for Young Investigators in Circulatory and Respiratory Health

**DOI:** 10.1155/2016/5260134

**Published:** 2016-02-29

**Authors:** Salvatore Mottillo, Pierre Boyle, Lindsay D. Jacobi Cadete, Jean-Lucien Rouleau, Mark J. Eisenberg

**Affiliations:** ^1^Divisions of Cardiology and Clinical Epidemiology, Jewish General Hospital-McGill University, Montreal, QC, Canada H3T 1E2; ^2^Lady Davis Institute for Medical Research, Jewish General Hospital-McGill University, Montreal, QC, Canada H3T 1E2; ^3^Department of Emergency Medicine, McGill University Health Center, Montreal, QC, Canada H4A 3J1; ^4^Department of Epidemiology, Biostatistics, and Occupational Health, McGill University, Montreal, QC, Canada H3A 1A2; ^5^Institute of Circulatory and Respiratory Health, Canadian Institutes of Health Research, Ottawa, ON, Canada K1A 0W9; ^6^Montreal Heart Institute, University of Montreal, Montreal, QC, Canada H1T 1C8

## Abstract

*Background*. Improving mentorship may help decrease the shortage of young investigators (graduate students, postdoctoral fellows, and new investigators) available to work as independent researchers in cardiovascular and respiratory health.* Objectives*. To determine (1) the mentoring practices for trainees affiliated with the Canadian Institutes of Health Research (CIHR), Institute of Circulatory and Respiratory Health (ICRH), (2) the positive attributes of mentors, and (3) the recommendations regarding what makes good mentorship.* Methods*. We conducted a survey and descriptive analysis of young investigators with a CIHR Training and Salary Award from 2010 to 2013 or who submitted an abstract to the ICRH 2014 Young Investigators Forum. Clinicians were compared to nonclinicians.* Results*. Of 172 participants, 7.0% had no mentor. Only 43.6% had defined goals and 40.7% had defined timelines, while 54.1% had informal forms of mentorship. A significant proportion (33.1%) felt that their current mentorship did not meet their needs. Among clinicians, 22.2% would not have chosen the same mentor again versus 11.4% of nonclinicians. All participants favored mentors who provided guidance on career and work-life balance. Suggestions for improved mentoring included formal mentorship, increased networking, and quality assurance.* Conclusion*. There is an important need to improve mentoring in cardiovascular and respiratory health.

## 1. Introduction

The Institute of Circulatory and Respiratory Health (ICRH) is one of 13 institutes of the Canadian Institutes of Health Research (CIHR). The mandate of the ICRH is very broad and encompasses a wide range of communities that conduct research on heart, lung, brain, blood and blood vessels, critical and intensive care, and sleep [1]. Recent data has shown that the relative growth in number of funded grants and awards within the ICRH's mandated research areas has been slower as compared with the other 12 institutes of the CIHR [2]. This lack of growth varies markedly according to the research community served within ICRH but is particularly evident within the realms of cardiovascular and respiratory research. Further evidence demonstrates that this slow growth is attributed to a declining number of young investigators applying for grants and awards. For the purpose of this study, the term “young investigator” will be used in the broadest sense, encompassing graduate students, postdoctoral fellows, and new investigators (defined as researchers within 60 months of their first academic appointment).

Mentorship is defined as a relationship in which a more experienced individual provides guidance and support in both the professional and personal aspects of career development to their mentee [2]. Mentors play a major role in the development and transition of young investigators to independent researchers and have been identified as a key priority by the ICRH [3]. Improving mentoring practices within research areas under the mandate of the ICRH may help increase the number of young investigators evolving into independent researchers. However, appropriate mentorship is challenging, due to the ever-increasing multidisciplinary nature of health research [4].

The literature demonstrates that mentoring practices are key to the development of young investigators [5, 6]. However, there is a need to identify which form of mentorship is most favorable for trainees in Circulatory and Respiratory Health. There are two major forms of mentorship: informal and formal [7, 8]. Informal mentors take on their role spontaneously, usually due to shared interests and chemistry with a young investigator [9]. Formal mentors are assigned to a particular mentee and follow a predefined structure when providing guidance and support [10]. Frequently, formal mentorship involves the use of a signed mentorship agreement, which serves as a contract outlining the roles and responsibilities of mentor and mentee.

Given the major role mentorship plays in scientific research, we conducted a pan-Canadian survey of young investigators under the mandated areas of the ICRH to evaluate current issues with mentorship in health research and how mentorship may be improved. Specifically, we sought to determine (1) the status of mentoring practices, (2) the personal and professional attributes of mentors that provide the most significant impact on training, (3) recommendations of mentees regarding what makes a good mentor and mentorship program, and (4) whether or not responses differed between clinicians and nonclinicians and between mentees in varying stages of their career.

## 2. Methods

### 2.1. Study Population

Via email, we invited young investigators (graduate students, postdoctoral fellows, and new investigators) to participate if they were funded through a CIHR Training and Salary Award within the period of 2010–2013 and if they named the ICRH as their primary CIHR institute. In addition, young investigators who submitted an abstract to the CIHR ICRH 2014 Young Investigators Forum were invited to participate. The Young Investigators Forum is a major training and education initiative of the ICRH, which provides young investigators the opportunity to showcase their research and develop professional networks with peers and mentors across Canada [11].

### 2.2. Study Procedures

We conducted a survey using self-administered questionnaires that young investigators were invited to complete online in English or in French. Eligible young investigators were sent an email invitation to our survey in April, 2014. Three reminder emails were sent out at one-week intervals, and the survey ran until May, 2014. Young investigators were excluded if they received funding outside of the date range, an email address was no longer valid, or if the survey submission was incomplete.

The survey was developed by the ICRH Advisory Board Working Group on Training, Mentoring, and Career Development, which consists of a panel of four health researchers from across Canada (see Acknowledgments). The survey was tested by these working group members and their research teams. The survey consisted of multi-categorical questions regarding funding information, mentorship status, topics/issues discussed with mentors, and ranking of personal and professional attributes of mentors (Online Appendix  A at Supplementary Material available online at http://dx.doi.org/10.1155/2016/5260134). There was also an open-ended question regarding recommendations of the young investigator as to what makes a good mentor and mentorship program. The questionnaire took approximately 15 minutes to complete.

### 2.3. Data Analysis

We conducted a descriptive analysis of the status of mentoring practices and the attributes of mentors of the young investigators surveyed. We stratified our results by gender, career stage, and whether or not the participant was a clinician. We also compared responders to nonresponders for these characteristics, as well as geographical location, in order to ensure that nonresponse bias did not threaten the validity of the results. Moreover, we summarized the answers to the open-ended question of our survey.

## 3. Results

### 3.1. Characteristics of Study Population

**Figure 1:**
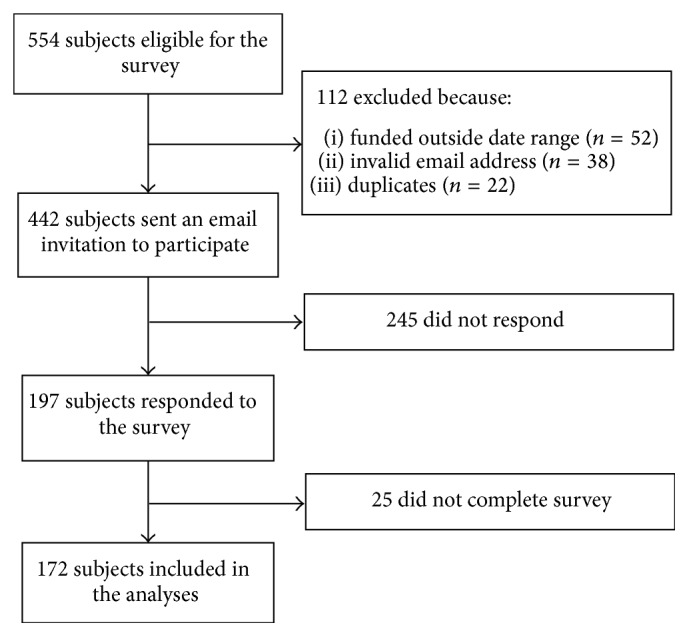
Flow diagram.

A total of 554 young investigators (graduate students, postdoctoral fellows, and new investigators) were identified as receiving funding through a CIHR Training and Salary Award and named the ICRH as their primary CIHR institute or submitted an abstract to the CIHR ICRH 2014 Young Investigators Forum (Figure 1). Of these, 112 young investigators were initially excluded for the following reasons: (1) funding was obtained outside the predefined date range of January 2010 to December 2013 (*n* = 52), (2) email address was no longer valid (*n* = 38), or (3) young investigators were counted as duplicates (i.e., received multiple sources of funding from the CIHR) (*n* = 22). Hence, after exclusions, 442 young investigators were sent an email invitation to participate in the survey, of whom 197 responded. Twenty-five surveys responses were excluded due to incomplete submission, leaving a total of 172 complete submissions that were included in the analyses (38.9% response rate).

Of survey respondents (Table 1), 47.1% were female (*n* = 81), and the majority were either in the doctoral stage of their career (*n* = 56; 32.0%) or the postdoctoral fellow stage (*n* = 55; 32.0%). The majority of respondents were nonclinicians (*n* = 123; 71.5%) and were affiliated with a hospital-based research institute (*n* = 72; 41.9%). Responses were obtained from all medical schools in Canada. Characteristics of nonrespondents were similar to those of respondents regarding gender, career stage, and geographic location. Specifically, 49.8% (*n* = 122) of nonrespondents were female, the majority (*n* = 180; 73.5%) were in the doctoral or postdoctoral fellow stage of their career, and the majority were from Ontario or Quebec (*n* = 152; 62.0%).

### 3.2. Status of Mentoring Practices

The majority of young investigators reported having their research supervisor as a mentor (*n* = 112; 65.1%) and the second most common form of mentoring consisted of a panel with multiple mentors (*n* = 63; 36.6%) (Table 2). Seven percent (*n* = 12) of respondents did not have a mentor. In most cases, the form of mentorship was informal (*n* = 93; 54.1%) or mixed (both informal and formal; *n* = 70; 40.7%). Only 12.8% (*n* = 22) of respondents had strictly formal mentoring. These forms of mentorship were not mutually exclusive; respondents may have checked off more than one form of mentorship, thereby causing the totals to add up to more than 100%. The type of mentor and the form of mentorship reported were similar across all career backgrounds, with the exception of new investigators, who tended to have a higher prevalence of peer and informal mentors.

A minority of respondents signed a mentorship agreement (*n* = 27; 15.7%). Only 43.6% (*n* = 75) of respondents had defined goals and 40.7% (*n* = 70) had defined timelines. Furthermore, less than half of respondents were involved in a mentoring program that had defined commitments or interactions with mentors. Among topics discussed with the mentor, research was the most prevalent (*n* = 159; 92.4%), followed by career guidance (*n* = 104; 60.5%). Over half (51.1%) of clinicians discussed administrative topics and 44.4% of clinicians discussed work-life balance compared to nonclinicians (35.0% and 27.6%, resp.). The majority of doctoral students (60.7%), postdoctoral students (78.2%), and new investigators (61.1%) reported being mentors for other trainees.

### 3.3. Appraisal of Current Mentoring Practices

Enthusiasm was the personal attribute of mentors having the most significant impact on training (*n* = 149; 86.6%) among all participants, irrespective of their career background (Table 3). Other personal attributes that were highly rated were approachability and honesty. The actions of mentors having the most significant impact were acting as a role model (*n* = 142; 82.6%), being accessible (*n* = 141; 82.0%), and actively listening (*n* = 140; 81.4%).

Approximately one-third (*n* = 57; 33.1%) of all participants felt that their current mentoring program did not meet their needs, with postdoctoral fellows having the highest percentage (*n* = 19; 34.5%) and Master's students having the lowest percentage (*n* = 2; 10.5%). However, only 25 participants (14.5%) reported that they would not choose the same mentor again. Clinicians and new investigators were the most likely to report not choosing the same mentor again (*n* = 10; 22.2% and *n* = 4; 22.2%, resp.).

### 3.4. Suggestions for Improved Mentoring Practices

Of the 27 participants with signed mentoring agreements, 15 (55.6%) found the agreements to be beneficial (Table 4). However, among the 142 participants without signed mentoring agreements, only 40 (28.2%) felt that such an agreement would be beneficial. Among topics that were currently not addressed in their mentoring practices, participants of all career stages primarily wished there would be more discussion regarding career guidance (*n* = 58; 33.7%), work-life balance (*n* = 55; 32.0%), and teaching/training (*n* = 46; 26.7%).

A total of 80 (46.5%) participants responded to an open-ended question asking for suggestions to improve the quality of mentoring practices. Key responses reflecting the suggestions made by survey participants were as follows.

Responses to the following question: “What suggestions would you provide for institutions looking to improve the quality of the mentoring experience for their trainees and new investigators?”

(1) Finding mentors:
   “Formal pairings might be beneficial for those not comfortable seeking out mentors.”
   “More networking opportunities with experienced mentors and trainees alike.”
   “Forcing a mentoring partnership is difficult because it depends on the personalities of those involved.”
(2) Type of mentor:
   “Multiple mentors can help balance viewpoints and provide a good support network.”
   “Have a senior and a junior mentor, if possible.”
   “It is important to have a mentor outside of the academic circle that one is constantly in contact with, in order to have a chance to learn about different opportunities within a field of research.”
(3) Form of mentorship and signed agreements:
   “Establish clear goals.”
   “Mentorship should be mandatory as it is crucial for the success of the new investigator.”
   “Mentorship should be formally implemented. Some mentors are not aware that mentoring is an important aspect of training.”
   “Provide strict guidelines and enforce them. It is becoming the norm that tenured professors do very little and this is culturally accepted within institutions.”
(4) Quality assurance and mentor/trainee relations:
   “An ombudsman who receives complaints and could investigate when the mentorship is not going well.”
   “Provide support for trainees to protect their intellectual property.”
   “Regular evaluations to gauge how well the relationship is going.”
   “Do not eliminate training/new investigator salary support grants. This non-institutional support provides the mentee with substantial leverage and respect that certain mentee-directed goals are met.”
   “Introduce an anonymous evaluation process of mentors.”
(5) Incentives for mentorship:
   “Need allocated time for mentorship.”
   “Without some incentive system for mentoring (other than altruism), I'm afraid that researchers will continue focusing on what gets them the next data/grant/paper.”
(6) Attributes of ideal mentor:
   “Strongly connected mentors.”
   “So much focus on the quantitative (the easy to measure) has left the important qualitative (hard to measure) out of the equation and we are likely making a big mistake.”
   “Hire investigators who are sincerely dedicated to mentorship, are good with people, and can act as capable managers - not just individuals with good publication records.”
   “Trainees need concrete help, such as funding, lab space, and equipment. Trainees struggle early in their career because those things are missing and extremely hard to get.”
(7) Mentorship guidelines and training:
   “There should be formal training on evidence-based mentorship and supervision.”
   “At the beginning of the training, there should be an information session about mentorship.”
   “New investigators could be provided with a mentorship booklet at their start.”
   “A Mentorship course or workshop for mentors/mentees - outlining roles & responsibilities”.

Participants reported that there is a need to improve mentoring practices in Canada, beginning with finding mentors for trainees. They indicated the need for more networking opportunities and formal pairings for those who may have trouble finding mentors on their own. Participants favored multiple mentors, including senior and junior investigators, as well as mentors outside one's academic institution.

Participants were unanimous in seeking more formal mentorship with clear goals and guidelines. Several participants reported having the impression that mentorship was not taken seriously enough at their institution and quality assurance through regular evaluations was suggested. Other participants mentioned the need for incentives for mentorship as well as providing guidelines for training. Participants suggested that enthusiastic investigators with a dedication to mentorship are preferable. Finally, participants suggested that mentors who provide help with funding (e.g., by providing funding or help with filling out funding applications), equipment, and career guidance are particularly beneficial.

## 4. Interpretation

Our study was designed to evaluate the current mentoring practices in the areas of health research under the ICRH mandate. We found that most young investigators (trainees and new investigators) had an informal form of mentorship with loose timelines and no clear guidelines. We also found that young investigators favored mentors who were enthusiastic and dedicated to teaching over a mentor's success and publication status. Approximately one-third of participants felt that their current mentoring program did not meet their needs. Suggestions for improved mentoring practices principally included more formal mentorship. Additionally, participants suggested having more networking opportunities with potential mentors, incorporating quality assurance practices and incentives for mentorship, and providing more emphasis on career guidance, work-life balance, and resources, including sources of additional help (colleagues and contacts) and sources of equipment or supplies.

### 4.1. Access to Mentors and Resources

It is widely accepted that mentorship is a crucial component to the development of young investigators [3, 8, 12]. Mentorship fosters important research skills in addition to a wide range of aptitudes needed for career development, such as networking, teaching, obtaining funding, and work-life balance. Seven percent of participants reported having no mentor; however, even participants with a mentor reported the need for more networking opportunities to meet potential mentors and to benefit from multiple mentors. Multiple mentors may be favorable as they can provide different perspectives and research styles that a trainee may follow. New investigators may be of help with the “hands-on” portion of training. In contrast, senior investigators may provide trainees with career guidance and much-needed resources, as documented in several reviews of mentoring practices [7, 13–17].

### 4.2. Informal versus Formal Mentorship

Our survey demonstrated that approximately one-third of participants felt that their mentorship program did not meet their needs. Participants indicated that the absence of formal mentorship was, at least in part, responsible for their dissatisfaction with their mentorship program. Only 12.8% of participants reported having formal mentorship and less than half of participants had defined goals and timelines for their research. Participants' stressed that mentoring practices need to be more formal, with some participants suggesting that structured or formal mentorship should be mandatory. Participants were numerous in requesting clear goals, guidelines, and timelines for mentorship. Furthermore, several participants stressed the need for institution-based mentorship programs in which formal training on mentorship, including booklets and workshops, would be provided to mentors and mentees. Such training would outline the roles and responsibilities of both mentors and mentees so that the quality of mentoring practices would be improved.

These results are in concordance with findings from recent studies demonstrating that formal mentorship is more likely to be associated with improved mentee satisfaction at all levels of research training [15, 18, 19]. While formal mentorship may promote more effective mentoring, other studies warn that formal assignment of mentors by a department chair may be unfavorable. Specifically, Straus and Sackett showed that formal assignment of mentors by a department chair was a highly unpopular linkage strategy among mentees as it led to artificial relationships [5]. Hence, mentees may benefit from choosing their mentor (especially if they already have contact with one) albeit within the context of a formal mentorship program with clear goals, guidelines, and timelines.

Furthermore, despite the push for more formal mentorship, only a minority of participants felt that a signed agreement with their mentor would be beneficial. Participants reported that such an agreement would not be helpful if there was no system in place to ensure that both mentors and trainees adhere to their responsibilities. These findings concur with previous studies by Straus and Sackett and by Rosenblum that emphasize the need for valuing and rewarding mentoring practices in order to give credence to signed agreements [19, 20]. There is a need for quality assurance of mentor/mentee relations, either through an ombudsman and/or anonymous evaluations. Having an evaluation system in place, as well as incentives, such as allocated time, a mentor criterion for promotion and/or tenure, or a decrease in other research responsibilities may encourage investigators to take on mentorship roles and to place added value to such roles.

### 4.3. Attributes of Mentors Providing the Most Significant Impact

Consistent with previous studies [14, 18, 21], our study found that mentors who were enthusiastic, accessible, and acted as role models had the most significant impact on the training of young investigators (trainees and new investigators). Young investigators seemed to value these attributes more than highly successful investigators with excellent publication records. Young investigators were in search of mentors who are truly dedicated to training young researchers and who can provide the time necessary for their development. Drawing on evidence from competitions for* Nature's* Awards for Creative Mentoring, Lee et al. showed that most mentors were nominated for their high level of enthusiasm and their availability for career and personal guidance [21].

Topics participants wished would be discussed more often revolved primarily around career guidance and work-life balance as opposed to research or clinical skills. Young investigators were searching for mentors who can guide them in choosing and searching for a career position, finding resources, and making personal life choices—all intangibles that are not easily learned in a book. In effect, the suggestions reported by mentees in our survey correlate with the definition of what constitutes mentorship, that is, a partnership in both personal and professional growth and development [3, 7].

### 4.4. Mentorship in Clinicians versus Nonclinicians

Clinician-scientists are critical for driving health research innovation in Canada given the unique opportunity they have to undertake key aspects of scientific research within the clinical setting [22]. Of concern, however, are their declining numbers [23]; some experts consider the clinician-scientist to be an “endangered species” [24, 25]. Given that clinician-scientists have to divide their time between clinical and research duties, high-quality mentorship may be particularly important for this population. Clinicians discussed more administrative topics (university or hospital policies) and work-life balance, likely owing to the multiple responsibilities that clinician-scientists need to coordinate [26]. Overall, however, our survey demonstrated few differences in mentoring practices or recommendations between clinicians and nonclinicians.

### 4.5. Mentorship at Different Career Stages

New investigators (within 60 months of their first academic appointment) were more likely to favor discussions on administrative topics (university or hospital policies) and work-life balance. These preferences are likely due to the large number of responsibilities accrued, both personally and professionally, at this stage in their career. Interestingly, a majority of participants at the doctoral, postdoctoral, and new investigator stages reported being a mentor for other trainees. Hence, many participants were already being handed mentorship roles at the doctoral level, at a career stage when they may not have fully developed the skills to be independent researchers or mentors. The great potential of these peer mentors should not be ignored. Early in one's career, mentorship training should be initiated and value should be placed on these mentoring practices so that these young investigators can take interest and motivation in continuing their mentoring practices as senior investigators.

## 5. Limitations

There are potential limitations to our study. First, the response rate of individuals contacted to participate in our survey was 38.9% despite the multiple reminders that were sent out. However, characteristics of nonrespondents regarding gender, career stage, and geographic location were similar to those of respondents. Second, survey answer options, such as “excellent” or “very good,” may have led to unclear data since these options may represent different meanings to different respondents. However, the inclusion of open-ended questions helped increase the internal validity of our survey [27]. Third, the survey is essentially limited to those in biomedical sciences or clinicians, and the sample size of certain subgroups was rather small, such that the robustness of findings in these subgroups is limited. Finally, as with all online surveys, there was a risk that participants did not fully understand the questions [28]. We reduced this limitation by creating a survey with simple language that could be completed within 15 minutes.

## 6. Conclusion

Our study examined the mentoring practices under the mandated areas of the ICRH across Canada. Survey respondents favored enthusiastic mentors who are dedicated to teaching, act as role models, and who can provide networking resources for their career development. Clinicians and new investigators favored mentors who provide guidance on work-life balance and administrative issues. The vast majority of participants had access to a mentor; however, a significant proportion of survey respondents felt that their current mentoring program did not meet their needs. Suggestions to improve mentoring practices principally encompass a push toward formal mentorship with clear goals, timelines, and quality assurance of mentorship programs. Furthermore, a large proportion of young investigators serve as mentors for other students and may benefit from their own mentorship training early on in their career. Future studies are needed to determine whether or not these findings are applicable to areas of health research outside the mandate of the CIHR Institute of Circulatory and Respiratory Health.

## Supplementary Material

Online Appendix A consists of the survey that was administered to young investigators funded through a Canadian Institutes of Health Research (CIHR) Training and Salary Award from 2010 to 2013 and that named the Institute of Circulatory and Respiratory Health (ICRH) as their primary CIHR institute. Young investigators who submitted an abstract to the ICRH 2014 Young Investigators Forum were also invited to participate in the survey.

## Figures and Tables

**Table 1:** Characteristics of participants in the ICRH survey.

Characteristic	Clinician versus nonclinician^*∗*^		Total (*n* = 172)
	Nonclinician (*n* = 123)	Clinician (*n* = 45)	
Women, %	45.5	48.9	47.1
Geographic location, %			
Alberta	12.2	17.8	13.4
Atlantic Canada	4.9	4.4	5.2
British Columbia	12.2	8.9	10.5
Manitoba	6.5	6.7	6.4
Ontario	35.0	37.8	37.2
Quebec	10.6	20.0	15.7
Saskatchewan	0.8	0.0	0.6
International	9.8	4.4	8.1
Missing	8.1	0.0	2.9
Current career stage, %			
Master's student	10.6	11.1	11.0
Ph.D. student	32.5	33.3	32.0
Postdoctoral fellow	36.6	22.2	32.0
New investigator^†^	4.9	26.7	10.5
No longer pursuing research	4.1	0.0	2.9
Other	11.4	4.4	10.5
Missing	0.0	2.2	0.6
CIHR theme, %			
Biomedical	74.8	22.2	60.5
Clinical	18.7	66.7	32.0
Health system services	0.8	6.7	2.3
Social, cultural, environmental, and population health	5.7	4.4	5.2
Current training/working environment, %			
Campus-based	34.1	24.4	31.4
Hospital-based	39.0	51.1	41.9
Mixed campus/hospital-based	22.8	24.4	23.8
Other	4.1	0.0	2.9

CIHR: Canadian Institutes of Health Research; ICRH: Institute of Circulatory and Respiratory Health. ^*∗*^4 participants did not specify whether or not they were clinicians. ^†^Within 60 months of first academic appointment.

**Table 2:** Status of mentoring practices.

Mentorship characteristics^*∗*^	Clinician versus nonclinician^†^		Career stage^‡^				Total (*n* = 172)
	Nonclinician (*n* = 123)	Clinician (*n* = 45)	M.S. (*n* = 19)	Ph.D. (*n* = 56)	Postdoctoral (*n* = 55)	New investigator^§^ (*n* = 18)	
Type of mentor, %							
Panel (multiple mentors)	38.2	35.6	31.6	44.6	27.3	44.4	36.6
Single mentor	10.6	13.3	21.1	3.6	10.9	27.8	11.0
Supervisor acts as mentor	72.4	44.4	94.7	73.2	80.0	16.7	65.1
Peer mentor	15.4	20.0	26.3	10.7	12.7	38.9	16.9
No mentor	5.7	8.9	0.0	3.6	1.8	5.6	7.0
Other	4.9	6.7	0.0	3.6	0.0	0.0	1.2
Missing	0.0	0.0	0.0	0.0	0.0	0.0	0.0
Form of mentorship, %							
Formal	14.6	8.9	21.1	16.1	10.9	5.6	12.8
Informal	56.1	46.7	42.1	41.1	58.2	77.8	54.1
Mixed	43.1	37.8	63.2	57.1	38.2	11.1	40.7
Mandatory	17.1	6.7	36.8	23.2	7.3	0.0	14.0
No mentor	4.9	8.9	0.0	1.8	3.6	5.6	16.4
Other	0.0	6.7	0.0	3.6	0.0	0.0	1.7
Missing	0.0	0.0	0.0	0.0	0.0	0.0	0.0
Topics discussed with mentor, %							
Administrative	35.0	51.1	36.8	30.4	34.5	66.7	38.4
Clinical	4.9	40.0	21.1	14.3	9.1	22.2	14.0
Research	93.5	91.1	94.7	98.2	96.4	94.1	92.4
Teaching/training	51.2	60.0	57.9	60.7	41.8	61.1	52.3
Work-life balance	27.6	44.4	42.1	28.6	30.9	44.4	32.0
Career guidance	59.3	66.7	42.1	66.1	60.0	72.2	60.5
None of the above	4.9	4.4	0.0	0.0	3.6	0.0	2.3
Signed mentoring agreement, %							
Yes	17.1	13.3	26.3	16.1	16.4	11.1	15.7
No	82.1	84.4	73.7	82.1	83.6	88.9	83.1
Missing	0.8	2.2	0.0	1.8	0.0	0.0	1.2
Current mentoring program involves, %							
Defined goals	45.5	42.2	57.9	50.0	45.5	27.8	43.6
Defined timelines	43.9	35.6	42.1	51.8	43.6	22.2	40.7
Defined commitments for the mentor	28.5	20.0	42.1	30.4	29.1	5.6	25.6
Defined commitments for the trainee	38.2	28.9	52.6	35.7	41.8	16.7	34.9
Defined frequency and nature of interaction with the mentor	23.6	28.9	42.1	28.6	16.4	22.2	24.4
None of the above	35.0	35.6	26.3	30.4	32.7	50.0	36.6
Missing	1.6	0.0	0.0	1.8	0.0	0.0	1.2
Participant is a mentor for other trainees, %							
Yes	68.3	57.8	36.8	60.7	78.2	61.1	65.7
No	28.5	42.2	57.9	37.5	21.8	38.9	32.0
Missing	3.3	0.0	5.3	1.8	0.0	0.0	2.3

M.S.: Master's student; Ph.D.: doctoral student. ^*∗*^Stratified by clinical background and career stage so that columns are not mutually exclusive. Rows are not mutually exclusive; respondents may have checked off more than one form of mentorship, thereby causing the rows (totals) to be >100%. ^†^4 participants did not specify whether or not they were clinicians. ^‡^24 participants did not specify their career stage or marked their career stage as “other.” ^§^Within 60 months of first academic appointment.

**Table 3:** Participant appraisal of current mentoring practices.

Mentorship practices^*∗*^	Clinician versus nonclinician^†^		Career stage^‡^				Total (*n* = 172)
	Nonclinician (*n* = 123)	Clinician (*n* = 45)	M.S. (*n* = 19)	Ph.D. (*n* = 56)	Postdoctoral (*n* = 55)	New investigator^§^ (*n* = 18)	
Personal attributes of mentor with most significant impact on training, no. rated as “excellent” or “very good,” %							
Approachable	82.9	84.4	84.2	85.7	85.5	83.3	83.1
Altruistic/generous	67.5	80.0	73.7	71.4	70.9	88.9	70.3
Enthusiastic	94.6	93.3	84.2	91.1	89.1	94.4	86.6
Compassionate	68.3	77.8	73.7	69.6	72.7	83.3	70.3
Nonjudgmental	68.3	66.7	78.9	71.4	67.3	72.2	68.0
Patient	72.4	71.1	73.7	76.8	72.7	77.8	72.1
Honest/sincere	82.1	84.4	78.9	87.5	85.5	88.9	82.6
Reliable	74.8	77.8	73.7	76.8	80.0	83.3	75.6
Actions and behaviour of mentor with most significant impact on training, %							
Accessible	82.1	80.0	78.9	83.9	83.6	83.3	82.0
Actively listens	82.1	86.7	84.2	85.7	87.3	83.3	81.4
Provides moral support	71.5	64.4	63.2	75.0	76.4	61.1	65.7
Addresses personal issues	48.0	48.9	47.4	51.8	58.2	38.9	64.0
Assists in defining/reaching goals	63.4	77.8	73.7	71.4	70.9	66.7	62.8
Acts as a role model	82.1	84.4	78.9	87.5	87.3	83.3	82.6
Assists in skills development	74.0	77.8	73.7	80.4	78.2	77.8	75.0
Monitors career progression	67.5	68.9	68.4	76.8	67.3	66.7	68.0
Assists in navigating the institution	54.5	66.7	57.9	58.9	52.7	72.2	57.6
Facilitates networking	61.8	73.3	57.9	64.3	69.1	66.7	64.5
Participant feels he/she is in the driver's seat in regard to current mentoring, %							
Yes	68.3	68.9	68.4	71.4	63.6	77.8	68.6
No	28.5	26.7	31.6	23.2	32.7	22.2	27.9
Missing	3.3	4.4	0.0	5.4	3.6	0.0	3.5
Current mentoring program meets the needs of the participant, %							
Yes	64.2	64.4	89.5	58.9	65.5	66.7	65.1
No	34.1	33.3	10.5	39.3	34.5	33.3	33.1
Missing	1.6	2.2	0.0	1.8	0.0	0.0	1.7
Participant would choose the same mentor again, %							
Yes	84.6	73.3	89.5	85.7	89.1	72.2	80.8
No	11.4	22.2	10.5	14.3	10.9	22.2	14.5
Missing	4.1	4.4	0.0	0.0	0.0	5.6	4.7

M.S.: Master's student; Ph.D.: doctoral student. ^*∗*^Stratified by clinical background and career stage so that columns are not mutually exclusive. ^†^4 participants did not specify whether or not they were clinicians. ^‡^24 participants did not specify their career stage or marked their career stage as “other.” ^§^Within 60 months of first academic appointment.

**Table 4:** Participant recommendations for improved mentoring practices.

Mentorship characteristics^*∗*^	Clinician versus nonclinician^†^		Career stage^‡^				Total (*n* = 172)
	Nonclinician (*n* = 123)	Clinician (*n* = 45)	M.S. (*n* = 19)	Ph.D. (*n* = 56)	Postdoctoral (*n* = 55)	New investigator^§^ (*n* = 18)	
Participants with a signed agreement find it beneficial, %^||^							
Yes	52.4	66.6	60.0	55.6	44.4	50.0	55.6
No	47.6	33.3	40.0	44.4	55.6	50.0	44.4
Missing	0.0	0.0	0.0	0.0	0.0	0.0	0.0
Participants without a signed agreement feel it would be beneficial, %^¶^							
Yes	26.5	30.8	14.3	31.9	28.3	37.5	28.2
No	70.6	64.1	85.7	66.0	69.6	62.5	70.4
Missing	2.9	5.1	0.0	2.1	2.2	0.0	1.4
Topics participants wish would be discussed that are not currently addressed, %							
Administrative	17.1	11.1	10.5	10.7	21.8	11.1	15.7
Clinical	8.1	8.9	15.8	5.4	7.3	11.1	8.7
Research	11.4	11.1	10.5	5.4	14.5	11.1	11.6
Teaching/training	29.3	20.0	26.3	25.0	40.0	11.1	26.7
Work-life balance	34.1	26.7	42.1	42.9	30.9	11.1	32.0
Career guidance	36.6	28.9	36.8	37.5	40.0	22.2	33.7
None of the above	36.6	40.0	47.4	26.8	34.5	44.4	38.4

M.S.: Master's student; Ph.D.: doctoral student. ^*∗*^Stratified by clinical background and career stage so that columns are not mutually exclusive. ^†^4 participants did not specify whether or not they were clinicians. ^‡^24 participants did not specify their career stage or marked their career stage as “other.” ^§^Within 60 months of first academic appointment.  ^||^ *N*_total_ = 27.  ^¶^ *N*_total_ = 142.

## Abbreviations

CIHR: Canadian Institutes of Health Research
ICRH: Institute of Circulatory and Respiratory Health.
