# Supplementary material for: A National Survey of Mentoring Practices for Young Investigators in Circulatory and Respiratory Health
Source: Can Respir J. 2016 Feb 29;2016:5260134. doi: 10.1155/2016/5260134 (PMC4904521; doi:10.1155/2016/5260134)
Supplement: Supplementary file 1 — Online Appendix A consists of the survey that was administered to young investigators funded through a Canadian Institutes of Health Research (CIHR) Training and Salary Award from 2010 to 2013 and that named the Institute of Circulatory and Respiratory Health (ICRH) as their primary CIHR institute. Young investigators who submitted an abstract to the ICRH 2014 Young Investigators Forum were also invited to participate in the survey. [file 5260134.f1.docx]

**ONLINE APPENDIX A. Survey**

**CIHR-Institute of Circulatory & Respiratory Health: Assessing the Current Trainee Mentoring Environment**

**General Information**

1. I am a:

*(Question Type: Drop-down Menu)*

- Male
- Female
- Prefer not to answer

1. Between 2010 and 2013, I was funded through new and/or continuing funding by CIHR as a: *(Please select all that apply.)*

*(Question Type: Tick Boxes)*

- Master’s student
- PhD student
- Post-doctoral fellow
- New Investigator (within 60 months of first academic appointment)
- Other: (Please explain. *(Provide text field to allow for elaboration.)* )

1. I am currently a:

*(Question Type: Multiple Choice)*

- Master’s student
- PhD student
- Post-doctoral fellow
- New Investigator (within 60 months of first academic appointment)
- I am no longer pursuing a research career.
- Other: (Please explain. *(Provide text field to allow for elaboration.)* )

1. My primary CIHR institute is the:

*(Question Type: Drop-Down Menu)*

- Institute of Aboriginal Peoples` Health (IAPH)
- Institute of Aging (IA)
- Institute of Cancer (ICR)
- Institute of Circulatory and Respiratory Health (ICRH)
- Institute of Gender and Health (IGH)
- Institute of Genetics (IG)
- Institute of Health Services and Policy Research (IHSPR)
- Institute of Human Development, Child, and Youth Health (IHDCYH)
- Institute of Infection and Immunity (III)
- Institute of Musculoskeletal Health and Arthritis (IMHA)
- Institute of Neurosciences, Mental Health, and Addiction (INMHA)
- Institute of Nutrition, Metabolism, and Diabetes (INMD)
- Institute of Population and Public Health (IPPH)

1. My secondary CIHR institute is:

*(Question Type: Drop-Down Menu)*

- Institute of Aboriginal Peoples` Health (IAPH)
- Institute of Aging (IA)
- Institute of Cancer (ICR)
- Institute of Circulatory and Respiratory Health (ICRH)
- Institute of Gender and Health (IGH)
- Institute of Genetics (IG)
- Institute of Health Services and Policy Research (IHSPR)
- Institute of Human Development, Child, and Youth Health (IHDCYH)
- Institute of Infection and Immunity (III)
- Institute of Musculoskeletal Health and Arthritis (IMHA)
- Institute of Neurosciences, Mental Health, and Addiction (INMHA)
- Institute of Nutrition, Metabolism, and Diabetes (INMD)
- Institute of Population and Public Health (IPPH)
- Not-Applicable

1. The CIHR Research Theme which best describes my research is:

*(Question Type: Multiple Choice)*

- Theme 1 (Biomedical)
- Theme 2 (Clinical)
- Theme 3 (Health System Services)
- Theme 4 (Social, Cultural, Environmental, and Population Health)

**Once a participant has made a selection, question 4b. will become available)*

6b. Other CIHR Research Themes that apply to my research include: (*Please select all that apply).*

*(Question Type: Tick-boxes)*

- Theme 1 (Biomedical)
- Theme 2 (Clinical)
- Theme 3 (Health System Services)
- Theme 4 (Social, Cultural, Environmental, and Population Health)
- No other CIHR themes are applicable to my research.

**A participant will not be permitted to select the same answer as was provided in question 4.*

**Training**

1. Which of the following best describes your academic background:

*(Question Type: Multiple Choice)*

- Non-clinician
- Clinician MD
- Clinician non-MD (e.g. nursing, pharmacy, physical therapy, etc.)
- Other: (Please explain. *(Provide text field to allow for elaboration.)* )

1. My current academic institution is:

*(Question Type: Multiple Choice)*

- Dalhousie University
- McGill University
- McMaster University
- Memorial University
- Northern School of Medicine
- Queen`s University
- Western University
- University of Alberta
- University of British Colombia
- University of Calgary
- University of Laval
- University of Manitoba
- University of Montreal
- University of Ottawa
- University of Saskatchewan
- University of Sherbrooke
- University of Toronto
- Other: (Please provide name of current academic institution. *(Provide text field to allow for elaboration.)* )

1. My current training/working environment is:

*(Question Type: Multiple Choice)*

- Campus-based
- Hospital-based Research Institute
- Mixed: I train/work at both campus-based and hospital-based facilities.
- Other: (Please explain. *(Provide text field to allow for elaboration.)*

**Mentoring**

*A process whereby an experienced, highly regarded, empathetic person (the mentor) guides another (usually younger or more junior) individual (the mentee) in the development and re-examination of their own ideas, learning, and personal and professional development. The mentor, who often (but not necessarily) works in the same organization or field as the mentee, achieves this by listening or talking in confidence to the mentee.*

Source: Standing Committee on Postgraduate Medical and Dental Education. Supporting Doctors and Dentists at work: an enquiry into mentoring. 1998.

1. I presently have access to the following forms of mentorship: *(Please select all that apply.)*

*(Question Type: Tick-Boxes)*

- I have a mentorship panel composed of several mentors.
- I have a single mentor.
- My supervisor acts as my mentor.
- I have a peer mentor.
- I do not have a mentor.
- Other: (Please explain. *(Provide text field to allow for elaboration.)* )

1. I would describe my current mentoring as: *(Please select all that apply.)*

*(Question Type: Tick-Boxes)*

- Formal (e.g., includes a signed agreement between mentor and mentee, regular meetings, and recurrent feedback)
- Informal (e.g., ad hoc meetings with mentor as needed or possible)
- Mixed: I currently have access to both formal and informal mentoring.
- Mandatory (e.g. required by my department/college for my degree program)
- I do not have a mentor.
- Other: (Please explain. *(Provide text field to allow for elaboration.)*)

1. What topic/issues do you currently discuss during meetings with your mentor? (Please check all that apply) *(Question Type: Tick-Boxes)*

- Administrative (e.g. changes to hospital/university, policies, etc.)
- Clinical (e.g. on call responsibilities, etc.)
- Research (e.g. publications, grantwritings, presentations, etc.)
- Teaching/training (e.g. undergraduate, postgraduate, etc.)
- Work-life balance
- Career guidance (e.g. individual career plan, CV, etc.)
- None of the above
- Other: (Please explain. *(Provide text field to allow for elaboration.)*)

1. What topic/issues do you wish were discussed during meetings with your mentor that are NOT currently being addressed? (Please check all that apply)

*(Question Type: Tick-Boxes)*

- Administrative (e.g. changes to hospital/university, policies, etc.)
- Clinical (e.g. on call responsibilities, etc.)
- Research (e.g. publications, grantwritings, presentations, etc.)
- Teaching/training (e.g. undergraduate, postgraduate, etc.)
- Work-life balance
- Career guidance (e.g. individual career plan, CV, etc.)
- None of the above
- Other: (Please explain. *(Provide text field to allow for elaboration.)*)

A **mentoring agreement** serves as a `contract` and is signed by the mentee and mentor(s). It serves to clearly outline the expectations, roles, and responsibilities of the mentee and mentors.

1. I have a signed mentoring agreement with my mentor(s).

*(Question Type: Yes or No)*

- Yes *(Participant to be directed to question 14b.)*
- No *(Participant to be directed to question 14c.)*

12b. Is the signed mentoring agreement beneficial/useful?

*(Question type: Yes or No)*

- Yes
- No
- Please explain your answer: *(Provide text field to allow for elaboration.)*

12c. Do you believe it would be beneficial to have such a signed mentoring agreement?

*(Question type: Yes or No)*

- Yes
- No
- Please explain your answer: *(Provide text field to allow for elaboration.)*

1. My current mentoring program involves: *(Please select all that apply.)*

*(Question Type: Tick-Boxes)*

- Defined goals
- Defined timelines
- Defined commitments/roles/responsibilities for the mentor.
- Defined commitments/roles/responsibilities for the mentee.
- Defined frequency and nature of interaction with mentor(s). (e.g. monthly face to face meetings).

1. My current mentoring program meets my needs as a trainee/new investigator.

*(Question Type: Yes or No)*

- Yes
- No
- Please explain your answer: *(Provide text field to allow for elaboration.)*

1. I believe I am `in the driver’s seat’ in regards to my current mentoring.

*(Question Type: Yes or No)*

- Yes
- No
- Please explain your answer: *(Provide text field to allow for elaboration.)*

1. I am a mentor for other trainees.

*(Question Type: Yes or No)*

- Yes
- No
- Please explain your answer: *(Provide text field to allow for elaboration.)*

***Questions 19-21 will use the following scale:***

1. *Excellent*
2. *Very Good*
3. *Moderate*
4. *Poor*
5. *I do not know.*
6. Please rate the following personal attributes of the MENTOR who had the MOST significant impact on your training between 2010 and 2013:

(*Question Type: Multiple Choice 1-5)*

- Approachable (e.g. friendly and easy to talk to)
- Altruistic/Generous (e.g. dedicates substantial time and energy to mentoring with little or no professional benefit)
- Enthusiastic (e.g. demonstrates passion for their job)
- Compassionate (e.g. able to understand the mentees perspective)
- Non-Judgemental (e.g. encourages a mentee to determine their own career path without forcing their ideas about the ‘best’ or ‘correct’ career path on the mentee)
- Patient (e.g. able to accept delays and difficulties without becoming annoyed)
- Honest/Sincere (e.g. offers opinions on both the likely successes and potential failures of a mentee)
- Reliable (e.g. keeps mentoring appointments and commitments)
- Additional Comments: *(Provide text field to allow for elaboration.)*

1. Please rate the following actions and behaviours of the MENTOR who had the MOST significant impact on your training between 2010 and 2013:

(*Question Type: Multiple Choice 1-5)*

- Accessible (e.g. easily reached either in person or via telephone, email, etc.)
- Actively listens
- Provides moral support
- Addresses personal issues (e.g. work-life balance, etc.)
- Assists in defining and reaching goals
- Acts as role model of academic excellence, ethical behaviour, and professional conduct
- Assists in skills development (e.g. scientific writing, effective communications, etc).
- Monitors career progression
- Provides assistance in navigating the institution
- Facilitates networking with others in the field

1. Please rate the following professional attributes of the MENTOR who had the MOST significant impact on your training between 2010 and 2013:

(*Question Type: Multiple Choice 1-5)*

- Successful and well-respected in their field
- Well-connected to additional resources (e.g. access to equipment, supplies, etc.)
- Well-connected to sources of additional help (e.g. colleagues and contacts)
- Additional Comments: *(Provide text field to allow for elaboration.)*

1. Beyond your primary mentor, what other environments have provided mentorship opportunities to facilitate your career development?

- University
- Hospital Research Institute
- Colleges (e.g. graduate studies, Medicine, etc.)
- Department
- Conferences
- None of the Above
- Other: (Please explain. *(Provide text field to allow for elaboration.*)

1. Would you choose the same mentor again?

*(Question Type: Yes or No)*

- Yes
- No
- Please explain your answer: *(Provide text field to allow for elaboration.)*

1. Other than your mentors, to whom do you go to for advice? (*Please check all that apply.)*

*(Question Type: Tick-Boxes)*

- Departmental Chair
- Graduate Chair/Coordinator of my department
- Senior Investigator within my department
- Senior Investigator within my academic institution
- New Investigator within my department
- New Investigator within my academic institution
- Other trainees
- Other: *(Provide text field to allow for elaboration.)*

1. What suggestions would you provide for institutions looking to improve the quality of the mentoring experience for their trainees and new investigators?

*(Question Type: Text field.)*

1. Additional Comments/Suggestions:

*(Question Type: Text field.)*
